# Stable disease after high dose interleukin-2 (HD IL-2) immunotherapy: observations on long term survival and clinical benefit of additional HD IL-2

**DOI:** 10.1186/2051-1426-2-S3-P88

**Published:** 2014-11-06

**Authors:** Howard L Kaufman, Sandra Aung, Michael Morse, Michael Wong, James Lowder, Gregory Daniels, David McDermott

**Affiliations:** 1 Rutgers Cancer Institute of New Jersey, New Brunswick, NJ, USA; 2 Prometheus Laboratory, San Diego, CA, USA; 3 Duke University, Durham, NC, USA; 4 Department of Medicine, University of Southern California, Los Angeles, CA, USA; 5 Prometheus Laboratory, San Diego, CA, USA; 6 Moores Cancer Center, San Diego, CA, USA; 7 Beth Israel Hospital Deaconess Medical Center, Boston, MA, USA

## 

Patients with stable disease (SD) following cancer treatment have traditionally not been considered responders. We, and others, have previously shown that SD is an important response criteria in cancer patients treated with HD IL-2 immunotherapy [1-5]. Here we summarize findings from 13 sites, including 97 mRCC and 170 mM patients enrolled in the retrospective cohort of a national HD IL-2 database (http://www.proclaimregistry.com). Patients in the database were enrolled between 2006-2011, in an era of immune checkpoint inhibitors and targeted therapies. In metastatic renal cell carcinoma (mRCC) the median overall survival (mOS) was not reached in patients assessed to have SD post HD IL-2 (Figure 1). The mOS was over 2.5 years in patients with stable disease with metastatic melanoma (mM). The median follow-up for both diseases was 3 years. We further sought to examine whether patients with SD after 1 course, received benefit with additional HD IL-2 treatment. In mRCC patients that did not respond to treatment but continued onto another cycle of HD IL-2, the mOS was not reached and was statistically significant from patients that stopped treatment (p = 0.0269) (Figure 2).

**Figure 1:**
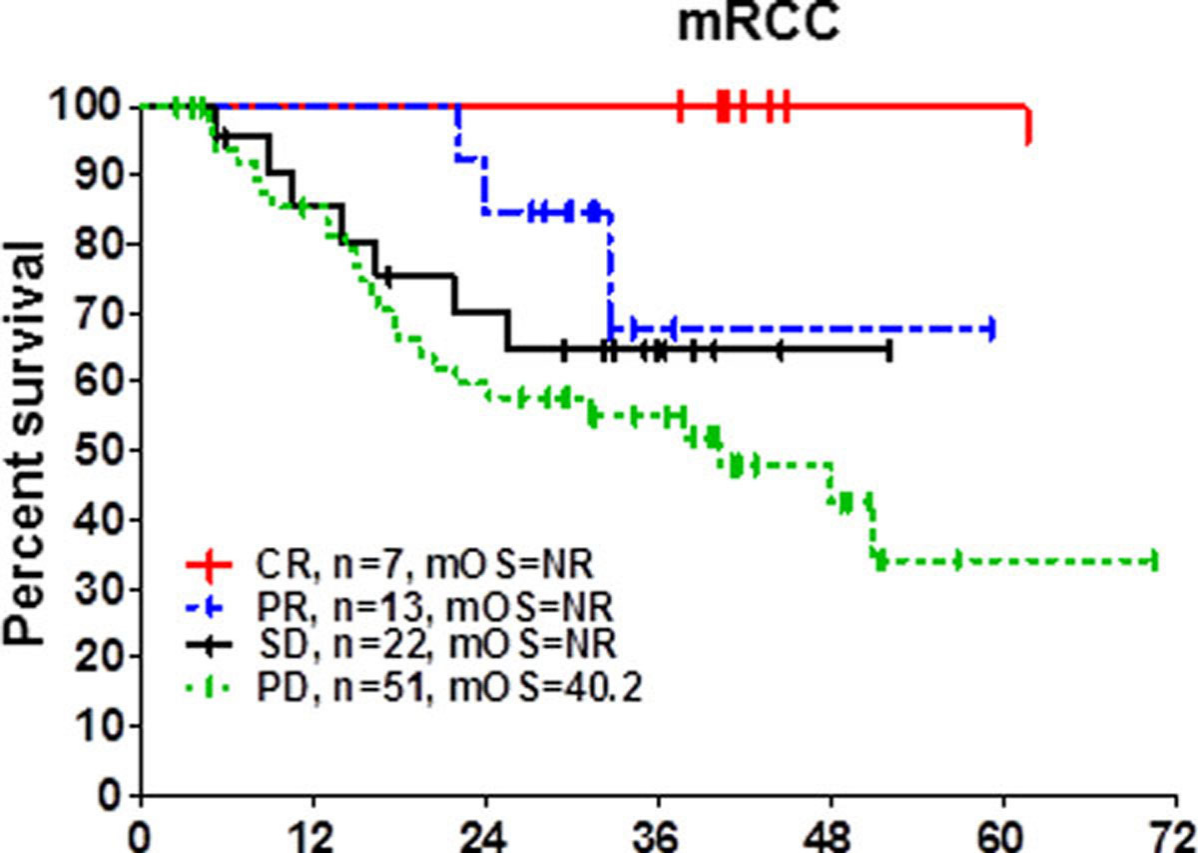
**Time Since First Dose of IL-2, Months**.

**Figure 2:**
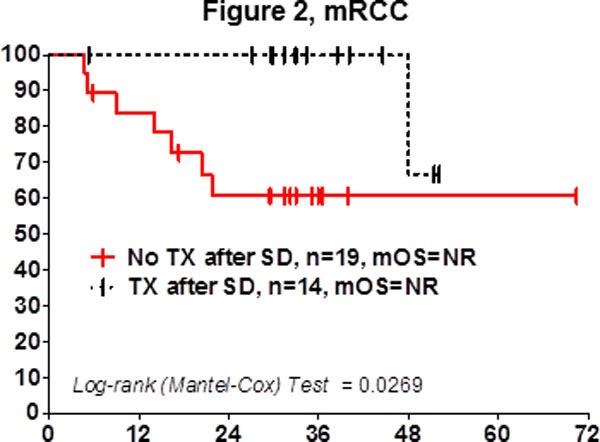
**Time Since First Dose of IL-2, Months**.

## Discussion

Unlike chemotherapy and targeted therapies, immunotherapeutics have the unique potential to achieve long lasting durable responses in cancer. The continual homeostasis between the immune system and the tumor requires constant immune pressure, and tipping the balance toward the immune system using immunotherapy may be important. Registries such as PROCLAIM^SM ^provide data which may guide the optimal sequencing of therapies and/or prioritization of randomized clinical trials [6].

## Conclusion

We conclude that stable disease is durable and should be considered a valuable end point. Consolidation of this response with additional HD IL-2 treatment following SD may be important.
